# Development of a Sensitive Detection Method for Alphaviruses and Its Use as a Virus Neutralization Assay

**DOI:** 10.3390/v13071191

**Published:** 2021-06-22

**Authors:** Christin Schmidt, Mario Perkovic, Barbara S. Schnierle

**Affiliations:** 1 Department of Virology, Paul-Ehrlich-Institut, Paul-Ehrlich Strasse 51-59, 63225 Langen, Germany; christin.schmidt@pei.de; 2 TRON (Translational Oncology at the University Medical Center), Johannes Gutenberg University Mainz, 55131 Mainz, Germany; Mario.Perkovic@TrOn-Mainz.DE

**Keywords:** chikungunya virus, Mayaro virus, Ross River virus, subgenomic promoter

## Abstract

Alphaviruses have a single-stranded, positive-sense RNA genome that contains two open reading frames encoding either the non-structural or the structural genes. Upon infection, the genomic RNA is translated into the non-structural proteins (nsPs). NsPs are required for viral RNA replication and transcription driven from the subgenomic promoter (sgP). Transfection of an RNA encoding the luciferase gene under the control of the sgP into cells enabled the detection of replication-competent chikungunya virus (CHIKV) or Mayaro virus (MAYV) with high sensitivity as a function of the induced luciferase activity. This assay principle was additionally used to analyze virus-neutralizing antibodies in sera and might be an alternative to standard virus neutralization assays based on virus titration or the use of genetically modified tagged viruses.

## 1. Introduction

Alphaviruses are arthropod-transmitted viruses of the *Togaviridae* family. Members of the Old World alphaviruses, such as chikungunya virus (CHIKV), Mayaro virus (MAYV), and Ross River virus (RRV), cause an acute infection with high fever, rash, myalgia, and polyarthralgia that lasts around 4–7 days, with viremia and high viral loads. It is only during this viremic phase that the virus can be detected in the blood by nucleic acid testing. This phase is followed either by convalescence or a symptomatic chronic phase, which can last for years, with arthralgia affecting multiple joints [1,2]. No vaccine against or treatment for alphavirus infections are available.

Alphaviruses have a single-stranded, positive-sense RNA genome of approximately 12 kb that contains two open reading frames encoding either the non-structural (nsP) or the structural proteins. Upon infection, the genomic RNA (gRNA) is directly translated into a non-structural precursor protein, which is processed by viral and cellular proteases. NsPs are required for viral RNA replication, which includes the synthesis of a minus-sense RNA from the gRNA, resulting in double-stranded (ds) RNA intermediates. The minus-sense RNA serves as a template for the gRNA and the subgenomic RNA (sgRNA), the synthesis of which is controlled by the subgenomic promoter (sgP). The structural proteins are translated from the sgRNA [3].

We have developed a sensitive assay to detect infectious alphaviruses by exploiting the alphavirus genome replication. Transfection of an RNA encoding the luciferase gene under the control of the CHIKV sgP into cells enabled the detection of replication-competent CHIKV or MAYV with high sensitivity as a function of the induced luciferase activity. In contrast, infection with the more distantly related alphavirus RRV did not induce luciferase activity. This assay principle was further used to analyze virus-neutralizing antibodies in sera and might be an alternative to standard virus neutralization assays based on virus titration or the use of genetically modified tagged viruses.

## 2. Materials and Methods

### 2.1. Cell Culture

All cells were cultured at 37 °C under 5% CO2. HEK 293T (CRL-1573) cells and Vero E6 cells (ATCC CRL-1586) were grown in Dulbecco’s modified Eagle medium (DMEM; Lonza, Verviers, Belgium), and BHK 21 (CCL-10) cells in Roswell Park Memorial Institute medium (RPMI; Biowest, Nuaille, France). All media were supplemented with 10% fetal bovine serum (PAA, Pasching, Austria) and 5% L-glutamine (200 mM; Lonza, Verviers, Belgium).

### 2.2. Human and Mouse Samples

Human naïve serum was obtained from the German Red Cross from volunteer blood donors. CHIKV antibody-positive plasma samples were obtained from Puerto Rican blood donors as well as from Brazilian patients that had been clinically diagnosed with and later tested for CHIKV infections. Plasma samples were obtained from Brazilian patients followed at the Viral Hepatitis Ambulatory/FIOCRUZ/Rio de Janeiro following IRB approval of study amendments. The approval date was 10 May 2016 (Fiocruz IRB ID: 0142/01) [4]. RRV plasma samples were obtained from Australian blood donors, following approval from the Australian Red Cross Blood Service Human Research Ethics committee [4,5].

Mouse serum was collected after infection of Balb/c mice with 1 × 10^6^ pfu MAYV (Animal license no.: F107/1036; Regierungspräsidium Darmstadt, Germany).

### 2.3. Reporter RNA Constructs

For the construction of reporter RNAs, the firefly luciferase or eGFP gene were inserted in a CHIKV transreplicon (TR-RNA-luc and TR-RNA-GFP). The plasmid encoding the CHIKV TR with structural genes for capsid and spike was generated by Thermo Fisher Scientific (Darmstadt, Germany) by assembling synthetic oligonucleotides and/or PCR products and insertion into the plasmid pOK (KanR). The ordered plasmid bears the PacI site, T7 promotor sequence (TAATACGACTCACTATAG), first 307 nt of the CHIKV, isolate LR2006 OPY1 [6], stop codon TAA, CHIKV sequence from AgeI site to the end of the 3′ UTR, followed by a segmented 100-nucleotide poly (A) tail interrupted by a short linker including an NdeI site (A30LA70, where L = GCATATGACT). The entire cassette was transferred via PacI and NdeI sites into the pST1 plasmid described elsewhere [7]. The plasmid was opened via HindIII and NheI for the insertion of reporter genes. Firefly luciferase and eGFP were amplified by PCR from plasmids encoding SFV-derived TR described before [8] and inserted by a Cold Fusion system (System Biosciences, Palo Alto, CA, USA). Prior to in vitro transcription, template plasmids were linearized using the type IIS restriction enzyme SapI, which generates an unmasked poly (A) tail. Synthesis and purification of RNA have been previously described [8].

### 2.4. Viruses

The wild-type CHIKV was a kind gift from Matthias Niedrig (Robert-Koch-Institut, Berlin, Germany) and was amplified on BHK-21 cells [9]. CHIKV-mCherry was amplified as described before [10,11] and frozen aliquots were stored at −80 °C. The MAYV strain TC652 was obtained from the Public Health England Culture Collections (ECACC No. 0906281v). The RRV T48 strain cDNA was a kind gift from Richard Kuhn [12]. The RRV cDNA was linearized by Sac I restriction and RNA was in-vitro-transcribed using the HiScribe SP6 RNA Synthesis Kit (according to the manufacturer’s protocol; NEB, Frankfurt/Main, Germany). Virus was produced as described for the CHIKV-mCherry. Viral titers (in pfu/mL) were determined from frozen aliquots by plaque titration on Vero E6 cells.

The modified vaccinia virus Ankara (MVA) was amplified on DF-1 cells as described before [13].

### 2.5. CHIKV Infection of TR-RNA-GFP-Transfected Cells

HEK 293T cells (0.8 × 10^6^ per well) were seeded onto a 6-well plate, incubated at 37 °C for 24 h, and counted. Subsequently, cells were transfected with 2 µg TR-RNA-GFP and CHIKV-mCherry was added at a multiplicity of infection (MOI) of 3 after 1 h at 37 °C. The cells were collected after 16 h, washed, and resuspended in 4% paraformaldehyde in PBS, and then analyzed by flow cytometry. At least 20,000 events were acquired with an LSRII instrument and analyzed with BD FACSDiva™ software (BD Biosciences, Heidelberg, Germany).

### 2.6. Detection and Titration of Alphaviruses by Monitoring Luciferase Activity

In total, 25,000 HEK 293T cells were seeded in a volume of 100 µL DMEM in white CELLSTAR 96-well plates (Greiner Bio-One, Frickenhausen, Germany). After 24 h incubation at 37 °C, cells were transfected with 100 ng TR-RNA-luc per well using Lipofectamine^®^ MessengerMAX™ (according to the manufacturer’s protocol; Thermo Fisher, Darmstadt, Germany). Subsequently, 100 µL of serial virus dilutions (CHIKV, MAYV, RRV, MVA; 10^2^–10^8^ pfu/mL) were added in triplicate after 1 h at 37 °C. After a further 16 h of incubation, BriteLite (PerkinElmer, Rodgau, Germany) substrate (50 µL) was added to each well, and the cells were incubated for 5 min at room temperature. The luciferase signal was detected as counts/s with a Tecan Spark reader (Tecan, Männedorf, Switzerland) for 1 s per well.

For the detection of CHIKV in spiked human sera, CHIKV was added to human naïve serum in virus dilutions ranging from 10^3^ to 10^5^ pfu/mL and was used as described above for diluted virus.

### 2.7. Monitoring of CHIKV Neutralization Activity by Luciferase Measurement

In total, 6000 HEK 293T cells were seeded in a volume of 20 µL DMEM in white CELLSTAR 384-well microtiter plates (Greiner Bio-One, Frickenhausen, Germany) using a Matrix Multichannel Equalizer Electronic Pipette (Thermo Fisher Scientific, Darmstadt, Germany). After 24 h incubation at 37 °C, cells were transfected with 20 ng TR-RNA-luc per well. Subsequently, 600 pfu CHIKV in 10 µL DMEM plus 10 µL diluted human plasma or mouse serum in DMEM was added to the cell. Final serum/plasma dilutions ranged from 10^−2^ to 10^−7^ (1:10 dilutions). The virus–serum/plasma mixtures were incubated in 96-U-well plates (Thermo Fisher Scientific, Darmstadt, Germany) at 4 °C for 30 min before addition to the 384-well plates in triplicate. After 16 h of incubation, 10 µL BriteLite substrate (PerkinElmer, Rodgau, Germany) was added to each well, and luciferase activity was detected with a Tecan Spark reader (Tecan, Männedorf, Switzerland).

### 2.8. Statistical Analysis

Area under the curve (AUC) values were determined using the GraphPad Prism 7.04 software (La Jolla, CA, USA). The mean values and standard deviations were calculated with Excel.

## 3. Results

### 3.1. Detection of Infectious Alphaviruses by sgP-Driven Reporter Gene Expression

**Figure 1:**
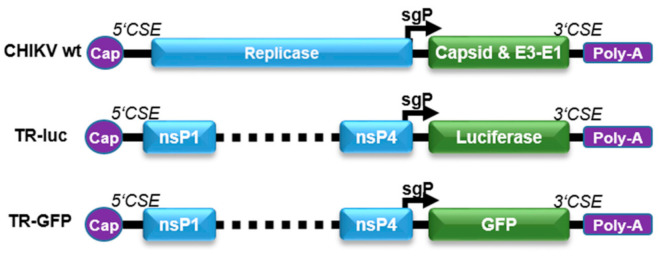
Reporter TR-RNA constructs. The reporter RNA constructs were designed by deleting the nsP1–4 proteins. The first 231 nt of nsP1, the last 1818 nt of nsP4, and the conserved sequence elements (5’CSE and 3’CSE) were retained in the RNA to allow monitoring of specific viral replication. The luciferase or GFP reporter gene was inserted after the sgP, replacing the structural proteins.

To be able to monitor CHIKV infection of target cells by the expression of reporter genes, the luciferase or green fluorescent protein (GFP) genes were placed under the control of the CHIKV sgP (Figure 1). The reporter plasmids (TR-luc and TR-GFP) were generated by deleting the non-structural proteins nsP1–4 (replicase), retaining only the first 231 nt of nsP1 and the last 1818 nt of nsP4, including the sgP sequence, as well as the alphavirus-conserved sequence elements (5’CSE and 3′CSE), which mediate specific viral replication [14]. The structural proteins were replaced by either the luciferase or GFP reporter gene, resulting in reporter RNA translation only in the presence of an alphavirus replicase in trans. After virus infection, the replicase is directly translated from the virus genome and subsequently amplifies the reporter RNA due to the presence of the alphavirus CSE. The sgRNA can be transcribed from this replicated minus-strand template by the viral replicase and the reporter proteins will be expressed in infected cells.

**Figure 2:**
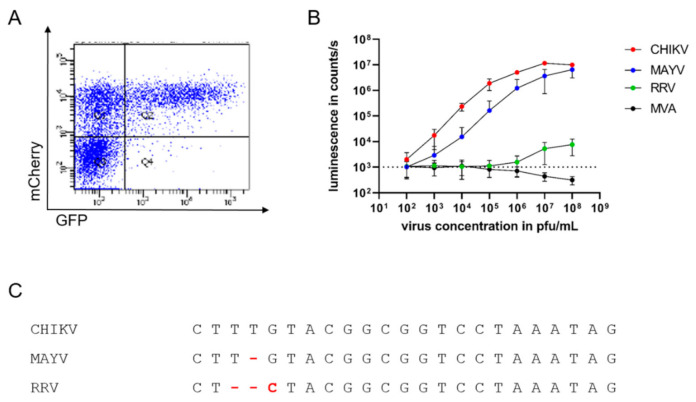
Specific and sensitive detection of infectious alphaviruses by sgP-driven reporter gene expression (**A**): HEK 293T cells were transfected with TR-RNA-GFP, infected with CHIKV-mCherry, and analyzed by flow cytometry after 16 h for GFP and mCherry fluorescence. The depicted graph is representative of three independent experiments. (**B**): TR-RNA-luc-transfected HEK 293T cells were infected with the alphaviruses CHIKV (red), MAYV (blue), and RRV (green), and the poxvirus MVA (black) at a virus concentration ranging from 10^2^ to 10^8^ pfu/mL. Luciferase signals were detected as counts/s after 16 h and compared to uninfected cells (dotted line). Depicted values are mean values ± SD of three independent experiments performed in triplicate. (**C**): Alignment of the sgP sequences of CHIKV, MAYV, and RRV.

This principle was first tested after transfection of the GFP reporter RNA and subsequent infection with a mCherry-expressing CHIKV [10]. Cells were analyzed after 16 h by flow cytometry. As expected, only cells infected with CHIKV-mCherry were also GFP-positive (Figure 2A upper right quadrant, Q2). In contrast, no cells expressing only green fluorescence were detected, indicating that reporter gene expression is dependent on CHIKV infection and the presence of replicase.

To further study the specificity and sensitivity of the assay and its suitability for high-throughput analyses, HEK 293T cells transfected with an RNA encoding luciferase under sgP control were infected with different amounts of CHIKV ranging from 10^2^ to 10^8^ pfu/mL. Compared to uninfected cells (indicated by dotted line), a luminescent signal could already be detected with 10^2^ pfu/mL CHIKV, corresponding to 10 pfu per well (depicted in red), demonstrating the high sensitivity of CHIKV detection (Figure 2B). The luminescent signal increased in a dose-dependent manner and reached a plateau at approximately 10^7^ pfu/mL (Figure 2B). To evaluate the specificity of the assay, the TR-RNA-transfected cells were infected with the alphaviruses MAYV and RRV and the poxvirus MVA. Similarly to CHIKV, infection with the closely related MAYV (depicted in blue) generated dose-dependent luminescent signals, although with lower signal intensity (Figure 2B). However, reporter protein expression after infection with the alphavirus RRV was only observed with virus concentrations of 10^7^ pfu/mL and above (depicted in green) and was absent after MVA infection of the cells (depicted in black).

Sequence comparison of the sgP shows the conservation of the sequence among the three alphaviruses, and changes in three base pairs influence the specificity of alphavirus replication (Figure 2C).

### 3.2. Detection of Viremia in Serum

**Figure 3:**
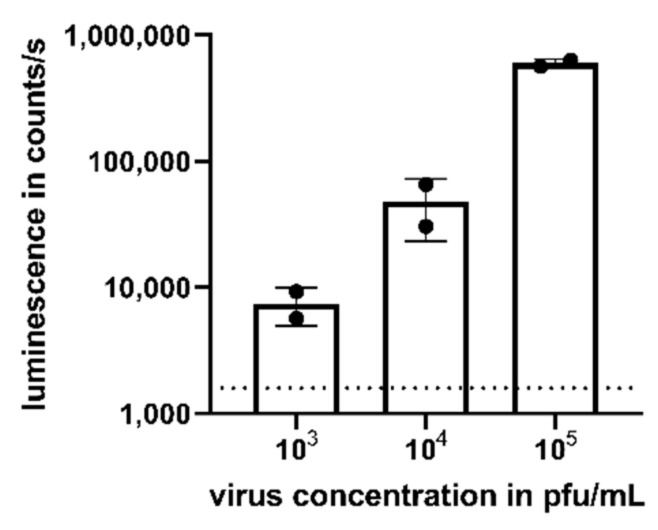
Detection of CHIKV viremia in human serum. Human serum was spiked with CHIKV with a concentration ranging from 10^3^ to 10^5^ pfu/mL prior to the addition to TR-RNA-luc-transfected HEK 293T cells. After 16 h, luciferase signals were detected as counts/s and compared to uninfected cells (dotted line). Depicted values are mean values ± SD of two independent experiments performed in triplicate.

We assessed the potential of the assay to detect viremia in the serum of CHIKV-infected patients. The mean viral load in asymptomatic and symptomatic patients has been reported as 3.4 × 10^3^ pfu/mL and 5.6 × 10^5^ pfu/mL, respectively [15]. Thus, we added CHIKV to human naïve serum in a virus concentration range from 10^3^ to 10^5^ pfu/mL to mimic viremic samples. Again, compared to uninfected cells (dotted line), a dose-dependent luminescent signal could be detected after 16 h for the studied virus concentrations (Figure 3).

### 3.3. Analysis of Neutralizing Antibodies

**Figure 4:**
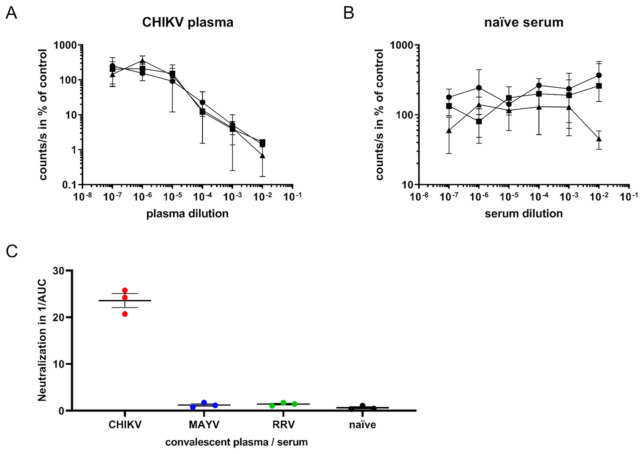
SgP-regulated luciferase expression to determine CHIKV-neutralizing antibodies. (**A**): CHIKV infection of TR-RNA-luc-transfected HEK 293T cells in the presence of three human plasma samples from convalescent CHIKV-infected patients at a dilution ranging from 10^−2^ to 10^−7^. Infection was determined as luciferase activity in counts/s and is presented as % of untreated control on a log scale. The values represent mean values ± SD of triplicate measurements. (**B**): CHIKV infection in the presence of human naïve serum. (**C**): Area under the curve (AUC) values were calculated using the GraphPad Prism 7.04 software. The reciprocal AUC values of neutralization assays with plasma from CHIKV- or RRV-infected patients and MAYV-infected mouse sera, or naïve serum samples, are depicted to illustrate CHIKV-specific neutralization activity.

Next, we evaluated whether the assay could be used to quantify virus-neutralizing antibodies from sera or plasma. For this purpose, 60,000 pfu/mL CHIKV, corresponding to 600 pfu per well (MOI 0.1), was mixed with plasma from convalescent CHIKV patients at dilutions ranging from 10^−2^ to 10^−7^ and incubated at 4 °C for 30 min [5]. The virus–sera mixtures were added to TR-RNA-luc-transfected 293T cells in 384-well plates. Luminescence was detected after 16 h. Dose-dependent neutralization of CHIKV by patient plasma was observed (Figure 4A). The lowest CHIKV plasma dilution (10^−2^) reduced the luminescence to only 1% of the untreated control infection, whereas the highest dilution (10^−7^) had no effect. Serum from CHIKV-naïve individuals did not inhibit the infection (Figure 4B).

To determine the specificity, the assay was performed with sera from MAYV-infected mice or plasma from RRV- or CHIKV-infected humans, as well as naïve sera. The reciprocal area under the curve (AUC) values, which reflect the neutralization capacity of the convalescent sera or plasma, were calculated and demonstrated that only plasma from CHIKV-infected patients specifically neutralized CHIKV (Figure 4C). No cross-neutralization of CHIKV with sera specific for the other alphaviruses MAYV and RRV was observed, demonstrating the high specificity of this virus neutralization assay.

## 4. Discussion

Alphaviruses, such as CHIKV, MAYV, and RRV, cause acute infections with high viral load viremia lasting around 4–7 days. During this time, the virus is usually detected in the blood by nucleic acid testing. However, nucleic acid tests determine the number of viral genomes and this does not necessarily resemble infectious virus titer. In order to verify the safety of blood products and vaccines, a stringent test for the presence of infectious agents is required.

Viruses coordinate their replication and transcription with specific sequence elements. Knowledge of these viral control elements can be used to establish simple screening methods for infectious viruses by measuring reporter protein expression, as has previously been shown for viruses including Influenza A, Herpes simplex, and Rubella [16,17,18]. In HIV research, the easy-to-perform TZM-bl assay has become the main endpoint neutralization assay used for the assessment of clinical trial samples. It measures antibody-mediated neutralization of HIV-1 as a reduction in HIV-1 Tat-regulated firefly luciferase (Luc) reporter gene expression [19,20].

Here, we exploited the alphavirus replication mechanism and constructed reporter RNAs expressing the luciferase or GFP gene under control of the CHIKV sgP. The single-stranded, positive-sense alphavirus RNA genome has two open reading frames encoding the non-structural or structural genes. The synthesis of the sgRNA by the viral replicase is controlled by the sgP. By replacing the structural genes with the reporter gene and deleting nsP1–3, viral replicase-dependent reporter templates were generated [14]. This assay principle has been used before to detect infectious Sindbis virus, another alphavirus, in mosquito or mammalian cells [21,22,23]. Deletion of only the highly conserved nsP4 sequence led to the construction of an alphavirus monitoring assay with broader specificity, allowing the detection of Sindbis virus, CHIKV, and Getah virus [22]. In contrast, the deletion of the less conserved nsP1–3 demonstrated here established a more specific alphavirus detection assay, able to detect as few as 10 CHIKV particles. Besides the detection of CHIKV, the closely related MAYV, but not the more distantly related RRV, was detected. Further closely related alphaviruses such as the O’nyong nyong virus might also be detected due to the similar sgP sequence.

This assay could be used for the detection of viremia in infected patients, and we were able to measure 10^3^ to 10^5^ pfu/mL CHIKV particles in human serum, which corresponds to the median viral loads observed in patients [15]. Nevertheless, in order to validate this assay for use in blood product safety, extensive assays with patient samples should be performed.

In contrast to our previous analyses with pseudotyped vector particles, this assay allows the analysis of authentic virus neutralization by antibodies with a high specificity [4]. However, it should be noted that human MAYV serum was difficult to obtain and therefore MAYV serum from infected mice was used in the study. Neutralization of CHIKV by convalescent MAYV patient serum cannot be excluded; however, the assay analyzes the neutralization capacity of antibodies, which is not species-dependent. This sensitive assay is suitable for high-throughput applications and is less time-consuming than traditional plaque reduction assays. Furthermore, TR-RNA transfection could be replaced by the generation of a stable cell line expressing the TR-RNA, analogous to HeLa-TZM-bl cells [19]. These cells would allow the detection of replication-competent CHIKV or MAYV, and CHIKV-neutralizing antibodies. Besides the quantification of CHIKV-neutralizing antibodies, this assay could also be used for the screening of virus inhibitors and could be adapted for other alphaviruses such as RRV by exchanging the sgP sequence.

## Data Availability

Data is contained within the article.
